# An ECG Stitching Scheme for Driver Arrhythmia Classification Based on Deep Learning

**DOI:** 10.3390/s23063257

**Published:** 2023-03-20

**Authors:** Do Hoon Kim, Gwangjin Lee, Seong Han Kim

**Affiliations:** Department of Intelligent Mechatronics Engineering, Sejong University, Seoul 05006, Republic of Korea

**Keywords:** ECG, EKG, electrocardiogram, ECG classification, ECG stitching, ECG concatenation

## Abstract

This study proposes an electrocardiogram (ECG) signal stitching scheme to detect arrhythmias in drivers during driving. When the ECG is measured through the steering wheel during driving, the data are always exposed to noise caused by vehicle vibrations, bumpy road conditions, and the driver’s steering wheel gripping force. The proposed scheme extracts stable ECG signals and transforms them into full 10 s ECG signals to classify arrhythmias using convolutional neural networks (CNN). Before the ECG stitching algorithm is applied, data preprocessing is performed. To extract the cycle from the collected ECG data, the R peaks are found and the TP interval segmentation is applied. An abnormal P peak is very difficult to find. Therefore, this study also introduces a P peak estimation method. Finally, 4 × 2.5 s ECG segments are collected. To classify arrhythmias with stitched ECG data, each time series’ ECG signal is transformed via the continuous wavelet transform (CWT) and short-time Fourier transform (STFT), and transfer learning is performed for classification using CNNs. Finally, the parameters of the networks that provide the best performance are investigated. According to the classification accuracy, GoogleNet with the CWT image set shows the best results. The classification accuracy is 82.39% for the stitched ECG data, while it is 88.99% for the original ECG data.

## 1. Introduction

Among abnormal heart diseases, atrial fibrillation (AF) is the most common persistent arrhythmia and occurs in 1–2% of the total population. AF increases the risk of stroke by a factor of five, and a fifth of all strokes result from arrhythmias [1]. Because arrhythmias appear and disappear suddenly, they are usually determined by long-term electrocardiogram (ECG) data. For an accurate ECG measurement, data must be acquired by attaching a 12-lead ECG measurement device to the patient, and during the measurement, the patient’s movement is not allowed to ensure the stable placement of the sensors. Furthermore, the diagnosis of arrhythmias is difficult because the symptoms appear and disappear, which makes it difficult to make a diagnosis by a scheduled ECG measurement in a hospital. In this respect, it would be meaningful if arrhythmias were detected through frequent ECG measurements.

In these days, individuals spend more time in their vehicles than before, creating new opportunities for integrating technologies such as virtual reality and augmented reality (VR/AR) and eye-tracking into vehicles [2]. AR technology, for example, can be used to provide drivers with real-time information about their surroundings, such as road conditions and traffic congestion. This information can be displayed on a heads-up display, allowing drivers to keep their eyes on the road while accessing important information [3]. Eye-tracking technology can be used to monitor drivers’ attention and alertness levels and provide feedback that can help drivers stay focused on the road [4]. An electroencephalogram (EEG) along with an ECG can be useful for driver safety. An EEG can be used to monitor driver fatigue or distraction levels, providing early warning signals that can help prevent accidents [5,6].

ECG recordings while driving can provide information about the driver’s physical and emotional state. For instance, ECG signals recorded during driving can indicate the driver's level of fatigue or stress as well as their cardiac health status, which may increase the likelihood of accidents. However, it would be very inconvenient if the 12-lead ECG electrodes were attached to the driver’s body in the vehicle to monitor the driver’s heart disease. Such a multi-lead ECG measurement is necessary for the accurate assessment of cardiac rhythm, but its usage is limited to long-term measurement.

Heart disease classification using single-lead ECG measurement devices has been studied by many researchers, and in recent years, the classification has been performed with the help of artificial intelligence (AI). Herry et al. proposed a heart rate classification scheme using synchronous transformation [7]. They studied machine-learning systems using the synchrosqueezing transform (SST)-derived instantaneous phase, R peak amplitude, R peak interval duration, etc. Miquel et al. presented a fully automatic and fast ECG arrhythmia classifier based on a simple brain-inspired machine-learning approach [8]. The presented classifier has low-demanding feature processing that only requires a single ECG lead. Awni et al. developed a deep neural network (DNN) to classify 12 rhythm classes using 91,232 single-lead ECGs from a single-lead ambulatory ECG monitoring device [9,10]. With the specificity fixed at the average specificity achieved by cardiologists, the sensitivity of the DNN exceeded the average cardiologist sensitivity for all rhythm classes. Mengze et al. proposed a robust and efficient 12-layer deep, one-dimensional convolutional neural network for classifying the five micro-classes of heartbeat types in the MIT-BIH Arrhythmia database [11]. Compared with the BP neural network, random forest, and other CNN networks, the model proposed in the study had better performance in accuracy, sensitivity, robustness, and anti-noise capability. Jonathan et al. developed an automatic classification algorithm for normal sinus rhythm (NSR), atrial fibrillation (AF), other rhythms (O), and noise from a single-lead short ECG segment (9–60 s) [12]. Unlike other studies using neural networks for ECG classifications, they used a signal quality index (SQI) along with dense convolutional neural networks. Mathews et al. achieved good performance for heart disease classification with a single-lead measurement ECG [13]. They also achieved good performance with simple features at low sampling rates. Wang et al. studied deep belief networks to extract and classify features from raw physiological data [14]. Zhang et al. proposed a one-dimensional convolutional neural network (CNN)-based heart disease classification [15], and Zubair et al. proposed an ECG classification system using CNNs that automatically learn appropriate feature representations from raw ECG data and ignore handcrafted features [16].

The methods proposed in previous studies assumed that the ECG acquisition occurred in a stable situation and that the required periods for data acquisition were 10 s or more. However, during an ECG acquisition while driving, data contamination can occur due to various artifacts, such as vehicle vibrations and bumpy road conditions. Therefore, it is difficult to acquire stable ECG signals in a moving vehicle, and using these contaminated data as inputs to a classifier can lead to erroneous results.

The objective of this study is to propose an ECG stitching scheme for the classification of drivers’ arrhythmias using a CNN. The proposed scheme includes selecting only stable signal segments from ECG measurements, which are then stitched together to generate full 10 s ECG data. The performance of the scheme is evaluated by comparing the accuracy of the arrhythmia classification model generated through transfer learning using the original ECG data and the stitched ECG data. The study consists of three steps: (i) data preprocessing, (ii) signal stitching, and (iii) transfer learning for ECG classification. In the data preprocessing step (Section 2.1), normalization, noise removal, and data flipping are performed. In the signal stitching step (Section 2.2), stable 2.5 s ECG segments are stitched together to generate the complete 10 s ECG signal. In the transfer learning step (Section 3 and Section 4), the stitched ECG signals are classified using different networks, and their parameters are studied to obtain the best results.

## 2. ECG Stitching

**Figure 1:**
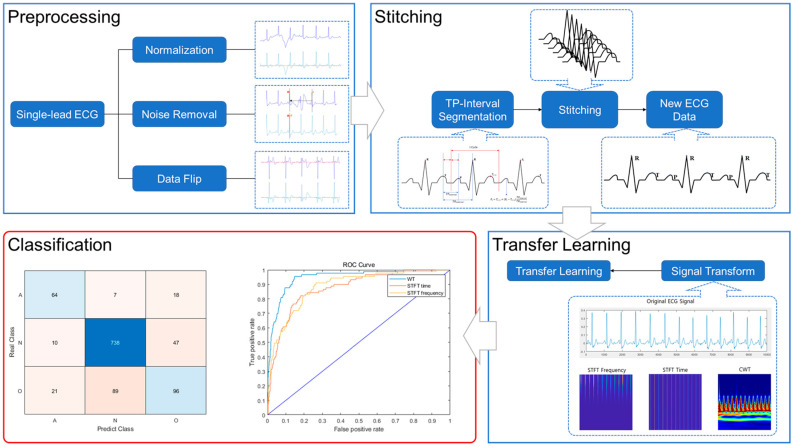
ECG stitching scheme for driver arrhythmia classification. Preprocessing: Data normalization, noise section removal, data flipping. Stitching: Reconstruction of ECG signal using TP interval segmentation. Transfer Learning and Classification: Prediction of driver arrhythmia.

The ECG stitching scheme proposed in this study consists of four processes. Figure 1 shows a schematic diagram that demonstrates the ECG stitching scheme for determining drivers’ arrhythmias. First, the driver’s ECG signal is collected through a single-lead ECG device mounted on the steering wheel of a vehicle. Second, the collected signal is preprocessed to remove noise caused by vehicle vibration and bumpy road surface conditions. Third, the preprocessed signal is divided into segments to determine and obtain a clean segment. Finally, the clean segments are merged into one ECG signal. The new ECG data generated in this way are used to determine the driver’s arrhythmias using a CNN model.

### 2.1. Preprocessing

**Figure 2:**
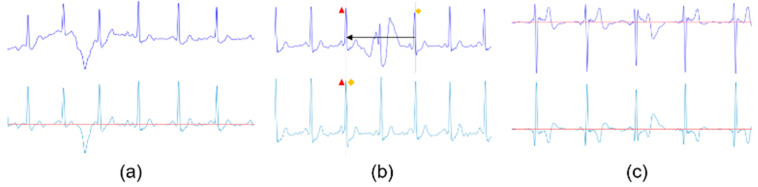
ECG preprocessing (**a**) normalization (**b**) noise section removal (**c**) data flip.

As mentioned, the driver’s ECG signal can be contaminated, and this leads to erroneous results in arrhythmia determinations. Prior to the stitching process, data preprocessing must be performed. As shown in Figure 2, the preprocessing used in this study is divided into three categories: normalization, noise section removal, and data flipping.

Normalization: When measuring an ECG signal, first-order median filters are used to remove baseline wander caused by the driver’s movement, breathing, and holding of the steering wheel [17]. This aims to connect two first-order median filters to estimate the baseline variability and subtract that estimate from the existing signal [18,19]. A low-pass filter is also used to remove high frequencies. Finally, a moving average filter is applied using a window with a size of 20 ms.

**Figure 3:**
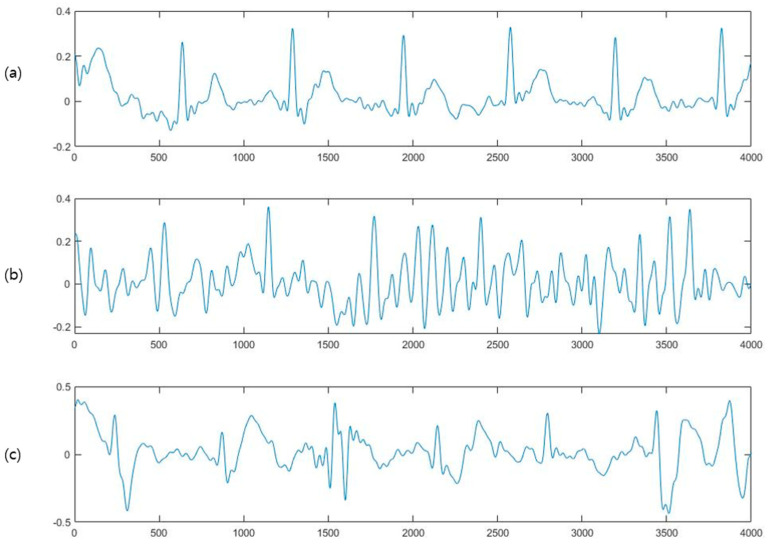
ECG data comparison (**a**) stable ECG data (**b**) ECG data when the steering wheel is squeezed (**c**) ECG data when in loose contact with the driver’s hands.

**Figure 4:**
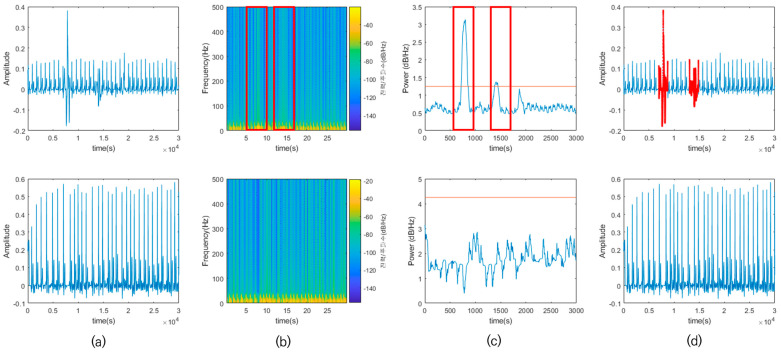
Normal ECG data with noise (top row) and ECG data of a patient with AF (bottom row) (**a**) normalized ECG data (**b**) spectrogram of ECG data (**c**) moving average of the sum of spectral power (**d**) ECG data to be removed (in red).

Noise section removal: The ECG data acquired via a single-lead ECG device have disadvantages such as that the data are vulnerable to noise generated when the driver grips the steering wheel. Figure 3a shows stable ECG data, while Figure 3b,c show the ECG data in which noise is generated by the driver’s gripping force. This type of noise can be removed by referring to the method of removing noise from the 300 Hz ECG signal performed in the study by Mukherjee et al. [20]. To remove the noise, a short-time Fourier transform (STFT) with a window size of 100 ms is used to make a spectrogram. As shown in Figure 4, the ECG signal with noise shows irregularly high energy, regardless of the QRS complex (Figure 4a,b). In the spectrogram, the sum of the spectral power is calculated for the range of 100 Hz or higher, which is higher than the general ECG signal. Then, a moving average with a 100 ms window size is calculated. As shown in Figure 4c, noise data have long and thick peaks, unlike those without noise. Finally, to remove the RR interval from the interval in which the spectral power exceeds the threshold, an adaptive threshold value that varies according to the energy map of the signal is required. Therefore, a value of 2.5 times the time series median of spectral power is a critical value, and the intervals between RR peaks and noise are removed from the ECG signal (Figure 4d).

Data flipping: When the electrodes are incorrectly placed on the steering wheel, the reversed ECG signal is acquired. This issue can be solved by finding the R peak of the ECG data and reversing the data if the average and median values of the R peak are below 0.

### 2.2. Signal Stitching

When ECG data are collected for more than 10 s at a time, the data usually come with noise. Therefore, this study proposes a method of dividing the cycles from the ECG data and storing them in a buffer. For this method, ECG signals are measured several times at short intervals, and the entirety of the 10 s ECG data period is generated by collecting and stitching only clean signals.

**Figure 5:**
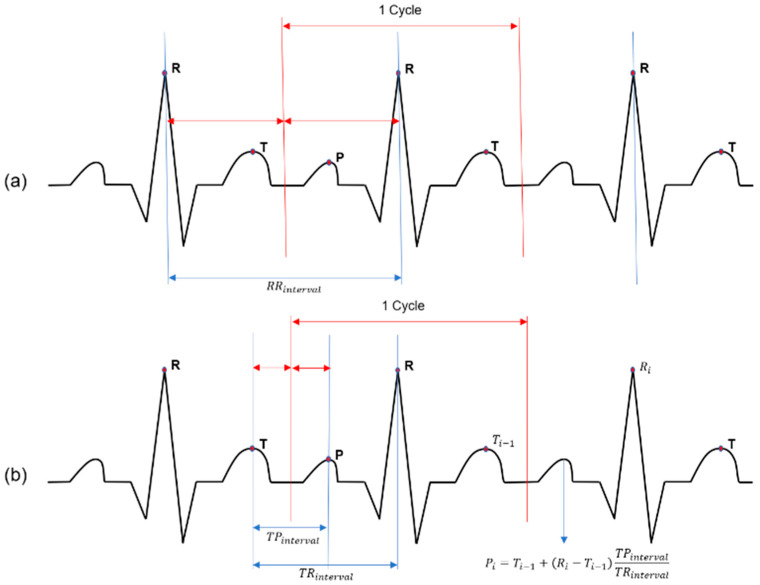
ECG stitching process (**a**) RR interval segmentation (**b**) TP interval segmentation.

For ECG stitching, this study focuses on using the RR interval in the processing of the ECG signals. First, as shown in Figure 5a, the R peaks are found in the collected data. If the central position between each R peak interval is set as the dividing point and the data can be divided based on the corresponding position, then one cycle can be obtained. When each cycle is collected in this way, only the R peaks are required for data stitching, so a high processing speed can be achieved. However, when a person has an arrhythmia with variable RR intervals or is in a situation where a fast heartbeat occurs, the splitting point has an unknown location, so it is not possible to obtain a complete cycle.

Therefore, the ECG stitching based on the RR interval was improved by applying the TP interval. As shown in Figure 5b, by setting a standard for dividing a cycle based on the T peak, which is the last point of a cycle, and the P peak, which is the first point of a cycle, data with a constant standard can be acquired. However, among the P, Q, R, S, and T peaks in ECG data, the most difficult to detect is an abnormal P peak [21]. Therefore, the TP interval segmentation method is divided into two cases. First, if the ECG peak points are found in the collected data, segmentation can be performed using the T and P peaks. However, if a P peak is not detected, they cannot be divided using the TP interval, so the P peak estimation method is used. The ratio of the existing TR and TP intervals was calculated using the P, R, and T peaks to predict a new P point using the average value of the ratio. Using the P and T peaks detected by applying the proposed method, a desired point can be divided based on the same criteria, even for an abnormal state, with the following equations.

(1) $${\mathrm{TP}}_{\mathrm{ratio}}=\frac{{\mathrm{TP}}_{\mathrm{interval}}}{{\mathrm{TR}}_{\mathrm{interval}}}$$

(2) $$P_{i}=T_{i-1}+\left(R_{i}-T_{i-1}\right){\mathrm{TP}}_{\mathrm{ratio}}$$

**Figure 6:**
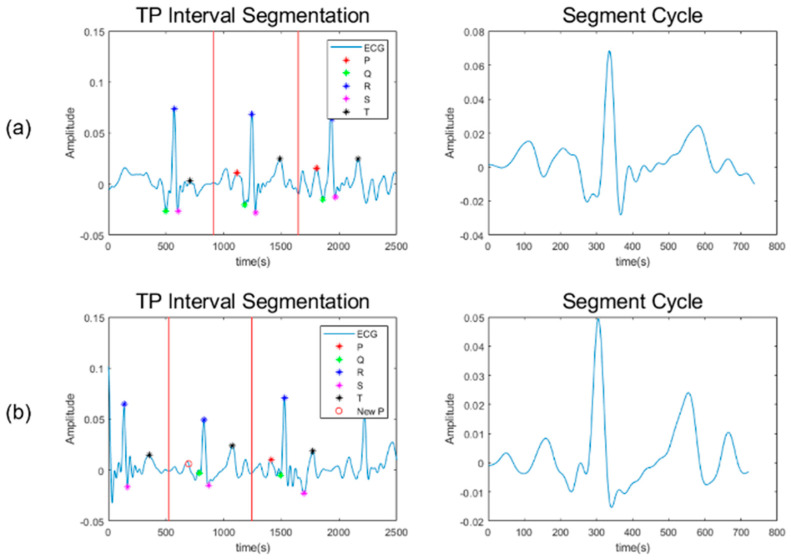
P peak detection for segmentation (**a**) When all P peaks are detected (**b**) When only one P peak is detected.

Through this method, TP interval segmentation can be applied to a person with an arrhythmia. Figure 6a shows the case when a cycle is normally divided using the central point between TP. As shown, all P peaks are found. However, in Figure 6b, the P peak is not detected due to an arrhythmia. In this case, a new P peak is detected using Equation (2), and the cycle is normally divided based on that point.

**Figure 7:**
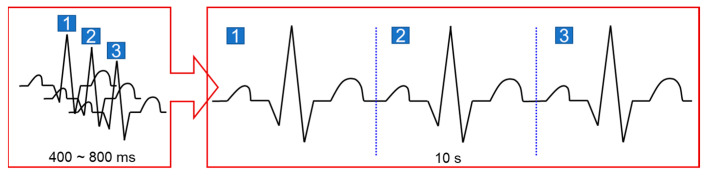
Generation of a new ECG signal from the segmented signals.

To effectively implement signal stitching in a moving vehicle, the acquisition time of an ECG signal must be as short as possible. The shorter the acquisition time, the smaller the possibility of data contamination. However, at least three R peaks are required to perform the TP interval segmentation. When the heart rate is between 60 and 70 bpm, one cycle has a length of 510–600 ms, and, accordingly, 1800 ms is required for three cycles, but this range only applies under normal conditions. If a driver has AF or other diseases, the RR interval becomes longer. In this case, it is difficult to obtain more than three R peaks. If three peaks or more are not obtained, the data cannot be used and should be discarded because the split point for data cannot be specified. To prevent this, data are collected by adding a spare time gap of 1.8 s, making it easier to obtain three or more R peaks. Based on the data measured for such a short time, the segmentation process is performed, and the resulting data are stored in a buffer. As shown in Figure 7, when a sufficient amount of data is collected in the buffer, the segmented ECG signals are stitched to generate a new 10 s ECG signal for arrhythmia classification.

**Figure 8:**
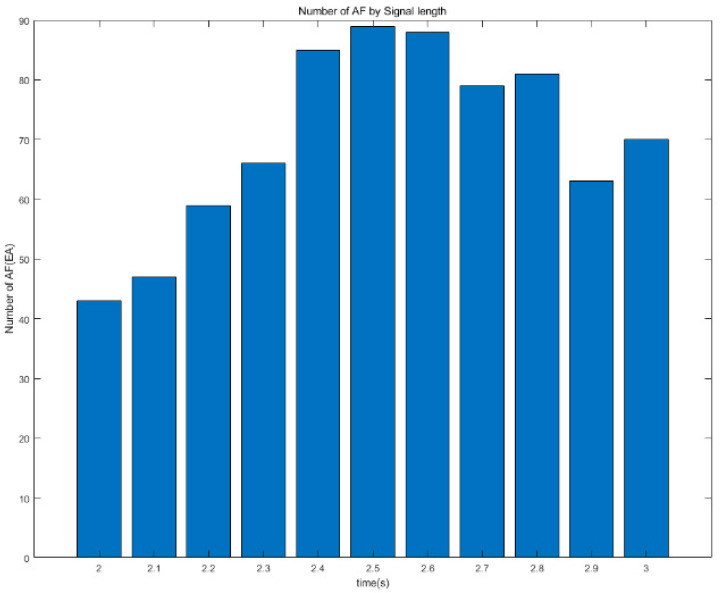
The number of AF classes by signal length.

For effective ECG stitching, it is very important to find the shortest time to collect the ECG signal for the segmentation. Experiments were conducted to determine the optimal time to improve the stitching performance by dividing the data collection time required for stitching by 0.1 s increments for 2–3 s. In the case of normal conditions or other diseases, according to the experimental results summarized in Table 1, the segmentation performs well, with no significant differences regardless of the time difference. However, comparing the results of AF by signal length, the number of stitched 10 s ECG signals varies. Comparing the AF graphs for each signal length in Figure 8, it can be seen that the number of stitched signals increases from 2 to 2.5 s; after reaching this maximum point, it then starts to decrease slightly. Therefore, considering that there is no major issue in data collection for the normal and other classes, the time at which the ECG signal has the highest number of AF obtained must be selected. The signal length used to stitch the ECG signal in this study is determined to be 2.5 s.

## 3. Convolutional Neural Network

CNNs have many applications, such as object detection, object recognition, image segmentation, face recognition, video classification, depth estimation, and image captioning [22]. In this study, CNN models, such as GoogleNet, SqueezeNet, ResNet, and DenseNet, were considered for transfer learning and used to classify arrhythmias.

### 3.1. Transfer Learning

Transfer learning is a method of learning a new classifier by using an existing neural network trained on a large dataset on a conceptually similar task without training from scratch. The CNN models used in this study were trained with over a million images that can be classified into 1000 objects. The trained neural networks learned different features representing different images. A pretrained neural network can be used as a starting point for new learning. It is usually easier and faster than training a neural network from scratch using randomly initialized weights. In this study, the network architectures were reused to classify ECG signals using transfer learning based on time series data images. The networks used for transfer learning in this study are GoogleNet, ResNet-101, SqueezeNet, and DenseNet-201.

GoogleNet uses a 1 × 1 convolution layer to reduce the number of feature maps, resulting in lowered computational needs. It contributed to reducing the computational resources of the model by using global average pooling to create a one-dimensional vector and by averaging each of the feature maps calculated from all layers [23].

ResNet-101 has 101 layers, while GoogleNet has 22 layers. Occasionally, a deeper network structure does not improve performance but rather degrades it. Thus, ResNet-101 created a shortcut that can add input values to output values using residual blocks. This concept is referred to as ResNet, meaning that the residual is minimized. After minimizing the residuals, the deeper the network structure, the better the performance [24].

SqueezeNet usually shows good performance with 50 times fewer parameters than AlexNet. Because the model is small and the required computational resource is low, the learning speed is fast. Due to the lower computational resource, information can be updated more frequently, which is good for handling constantly changing information. Furthermore, due to the small network size, even if it is installed in an embedded system, it does not impose too much of a load on the system. SqueezeNet shows good performance without the FC layer [25].

DenseNet also shows good performance with fewer parameters than ResNet. DenseNet connects the feature maps of all layers. Unlike ResNet, when concatenating, the size of the feature map is the same. Since the number of channels may increase if you continue to connect feature maps, the number of feature map channels in each layer is very small. DenseNet has a feature that shows the same performance with fewer training parameters compared to ResNet [26].

For transfer learning using these four networks with different characteristics, this study modified the last layers. In SqueezeNet, unlike the other three networks, the last layer is a 1 × 1 convolution layer. In this case, the number of convolution layer filters is the same as the number of classes (three in this study). For other networks, the last layer with learnable weights becomes the FC layer. Similarly, the number of output values of the FC layer was changed to a new FC layer equal to the number of classes. The final classification layer was then changed to a new layer and used for training.

### 3.2. Evaluation Matrix

In this study, the performance of a CNN model using transfer learning was evaluated by a confusion matrix, as in Table 2. The following ratios were computed for the model in the confusion matrix of the classifier trained to discriminate arrhythmias.

True positive (TP): Predict AF for AF class.

False positive (FP): Predict AF for a different class.

True negative (TN): Predict correctly for a different class.

False negative (FN): Predict a different class for AF class.

Accuracy: A measure of how closely the classifier is correct for the entire model, which is calculated by the following equation.

(3) $$\mathrm{Accuracy}=\frac{\mathrm{TP}+\mathrm{TN}}{\mathrm{TP}+\mathrm{TN}+\mathrm{FP}+\mathrm{FN}}$$

Precision: A measure of whether the classifier is correctly classified in each class, which is calculated by the following equation.

(4) $$\mathrm{Precision}=\frac{\mathrm{TP}}{\mathrm{TP}+\mathrm{FP}}$$

Recall: A measure of whether a given class is detected well by other classes, which is calculated by the following equation.

(5) $$\mathrm{Recall}=\frac{\mathrm{TP}}{\mathrm{TP}+\mathrm{FN}}$$

F1 score: This is a result value that shows the concepts of precision and recall at the same time using the harmonic mean. Therefore, the F1 score is calculated by the following equation.

(6) $$F1\;\mathrm{score}=\frac{2\times \mathrm{Precision}\times \mathrm{Recall}}{\mathrm{Precision}+\mathrm{Recall}}=\frac{2\mathrm{TP}}{2\mathrm{TP}+\mathrm{FP}+\mathrm{FN}}$$

Receiver operating characteristic (ROC) curve: The ROC curve and area under the ROC curve (AUC) are used to evaluate the performance of the proposed model. The ROC curve is used to compensate for the shortcomings in accuracy when the distribution for each class is different. In particular, it is usually used to compare deep learning models. The larger the AUC, the more stable the prediction and the better the model.

## 4. Training Setup

All experiments in this study were conducted on a system with an Intel Core i9 10900X processor, 128 GB RAM, and NVIDIA GeForce RTX 3090.

### 4.1. Dataset

The dataset used in this study consists of 8528 single-lead ECG data from the PhysioNet Computing in Cardiology Challenge 2017 [27]. The dataset has ECG signals between 9 and 60 s in length and consists of four classes: AF, normal, other rhythms, and noise. In this study, the training data were used by up sampling the data from 300 Hz to 1000 Hz.

### 4.2. Time–Frequency Analysis

A CNN model specializing in visual information data processing uses a multi-channel input layer to input RGB color information. This structure is utilized for time series data analysis to realize performance that cannot be achieved through conventional statistical analysis methods. In this study, the continuous wavelet transform (CWT) and short-time Fourier transform (STFT) were introduced to generate images from ECG data in the form of time series.

STFT divides a long signal that changes with time into short time units, applies the Fourier transform, and identifies which frequencies exist in each time interval. If a signal is divided into short time units, it is easier to know which frequencies exist at what time, and if the signal is divided into long time units, it is easier to know which frequencies exist within that time. The smaller the size of the window dividing the signal, the better the time resolution, and the larger the size, the better the frequency resolution. Since it is impossible for STFT to improve both time and frequency resolutions, it generates images for two cases with both resolutions. To calculate the time-dependent spectrum of the signal, the ECG signal is divided into 50 overlapping segments, and a window is applied to each segment using a Hann window. After calculating the STFT, these transforms are combined to create a matrix. First, a matrix with a time resolution of 125 is generated by dividing an ECG signal with a frequency of 1000 Hz by a divisor that varies with the signal length. Second, to improve the time resolution, a window segment length of 3 ms is specified, and the time resolution is calculated to create a matrix with a time resolution of 5000. Finally, a matrix with a high-frequency resolution and a matrix with a high-time resolution are obtained.

CWT increases the time resolution and decreases the frequency resolution for high-frequency domain signals. However, for a low-frequency domain signal, the frequency resolution is increased and the time resolution is decreased. While the STFT gives up either frequency resolution or time resolution, CWT is effective in time–frequency analyses. CWT was applied to convert the time–frequency representation of the ECG data into a scalogram.

**Figure 9:**
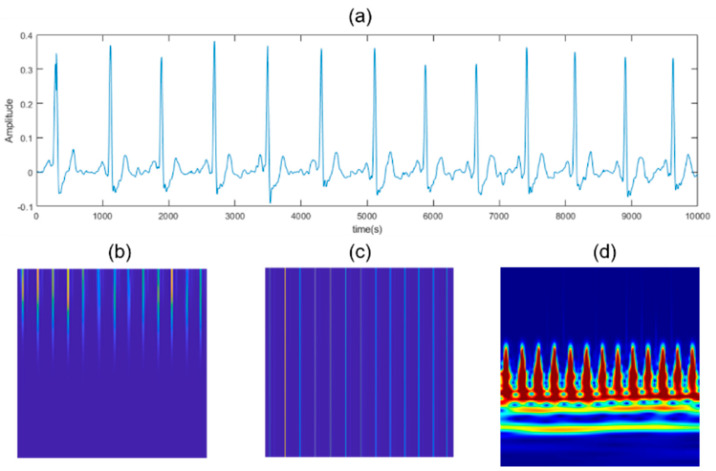
Time–frequency analysis on ECG data (**a**) ECG data (**b**) STFT high-frequency resolution (**c**) STFT high-time resolution (**d**) CWT.

To improve the computational efficiency when analyzing multiple signals at a given time frequency, the filter bank was precomputed once, and this filter was used as the input for the next process. If CWT is applied to the entire ECG signal using the calculated filter, it can be visualized in the time and frequency domains. However, since the converted spectrum cannot be converted into an image form because it comes out as a complex vector, the resulting value must be converted into a real number form. A matrix of real numbers can be obtained by taking the absolute value of the spectrum using the magnitude of a complex number that represents only the magnitude as the vector length from the origin to the complex value as it is plotted on the complex plane. The size of the matrix and complex number calculated in STFT and CWT is scaled to a range of 0–1, and this value is first converted to grayscale in the form of an 8-bit unsigned integer. Assigning a color map to the converted array creates an image with RGB values, as shown in Figure 9. The image was resized to fit the network size to 227 × 227 × 3 for SqueezeNet and 224 × 224 × 3 for other networks.

### 4.3. Training Parameters

#### 4.3.1. Test Dataset

This study uses PhysioNet data for the test dataset to validate the ECG stitching scheme proposed in this study. The PhysioNet data were divided into training, validation, and test datasets. To create the test dataset, the entire dataset was segmented into 2.5 s intervals, and only complete 10 s data segments created using the TP interval segmentation were selected as test data. The test data consists of 1090 samples, comprising 89 samples for AF, 795 samples for normal rhythms, and 206 samples for other rhythms.

#### 4.3.2. Training Dataset

To augment the training data from the PhysioNet data, the ECG signals with a length between 30 and 60 s were divided into 10 s groups. Through this augmentation, the number of training data records increased about three times, from 7438 to 20,475. For the holdout validation, the ratio of training data to validation data was set to 8:2, and the dataset was divided into 16,380 and 4095.

#### 4.3.3. Initial Training Options

Initial training parameters were set to provide the best prediction results. To minimize the loss function during network training, this study initially used the stochastic gradient descent with momentum (SGDM) optimizer. The SGDM evaluates the slope of the loss function at each iteration and updates the descent algorithm weights. After the SGDM was selected for the initial optimizer, the adaptive moment estimation (ADAM) was also considered to select the optimizer that provides the best performance. In addition, the initial learning rate used for training was set to 10^−4^ to slow the learning of the transferred layer that was not yet fixed. During training, the initial minibatch size was set to 64.

## 5. Results

### 5.1. Training Results

#### 5.1.1. Training

Experiments were conducted to select the best optimizer for arrhythmia classification between SGDM and ADAM. After training and comparing 12 models with four types of networks, as shown in Table 3, the model with the best performance was selected. The model selection aimed to achieve high validation accuracy and test accuracy, a small difference between the two levels of accuracy, and a small loss. As shown in Table 3, the network training results showed an accuracy between 76.8% and 82.39%, and the difference in accuracy between networks for the image set obtained through CWT was not significant. In addition, the network using the ADAM optimizer showed higher accuracy than the network using the SGDM optimizer for all networks. In the image set obtained through high-frequency-resolution STFT (STFT_F), GoogleNet and ResNet showed high accuracy when the ADAM optimizer was used. However, in terms of the difference in accuracy, SqueezeNet showed a difference of less than 1%. The overall performance of the STFT_F image set was lower than that of the other sets. Finally, the image set obtained through high-time-resolution STFT (STFT_T) showed that the performance of the model obtained through the SGDM optimizer was 3–6% lower than that of ADAM. In addition, the difference between validation and test accuracy was more than 3%. In the case of ADAM, all three models except DenseNet showed more than 80% accuracy, and the loss was small.

For arrhythmia classification, validation accuracy and test accuracy were higher with the ADAM optimizer, regardless of the network type and image set, and the difference between validation accuracy and test accuracy was relatively small. In addition, the loss was also relatively small. Therefore, since the ADAM optimizer was the best for all network models and arrhythmia classifications, ADAM was selected for the determination of training parameters.

#### 5.1.2. Networks

With the ADAM optimizer, the performance of the four network models with two minibatch size values was compared. For the CWT image set, the best validation accuracy and loss were achieved with a minibatch size of 64. The gap between validation accuracy and test accuracy was less than 0.6%, which meant that there was no overfitting. Rather, the overall performance decreased by more than 1% when the batch size was increased. For the STFT_F and STFT_T image sets, there were no significant differences in the minibatch size. For the STFT_F, GoogleNet and ResNet showed better performances at a minibatch size of 64, while better performances were found at a minibatch size of 128 in other networks. Similarly, in the case of STFT_T, GoogleNet and SqueezeNet showed better performance at a minibatch size of 64, while other networks did so at a minibatch size of 128. The performance of all networks is given in Table 4.

#### 5.1.3. L2 Regularization Rates

The four network models were also trained by adjusting the L2 regularization rate to three cases. Table 5 shows the performance of the network models when the L2 regularization rate was changed in three cases: 10^−4^, 10^−4^, and 0. For GoogleNet, the L2 regularization rate of 10^−4^ gave the best performance in CWT and STFT_F image sets, while the best performance for the others was provided when ResNet had 0 and DenseNet had 10^−4^, respectively. In the case of SqueezeNet, a model with an L2 regularization rate of 0 in CWT and 10^−4^ in STFT_F showed good performance. However, in the STFT_T image set, all models with an L2 regularization rate of 10^−4^ provided the best performance, regardless of the minibatch size. In this way, for the 12 models in each image set, the models with the best performance were selected.

### 5.2. Test Results

**Figure 10:**
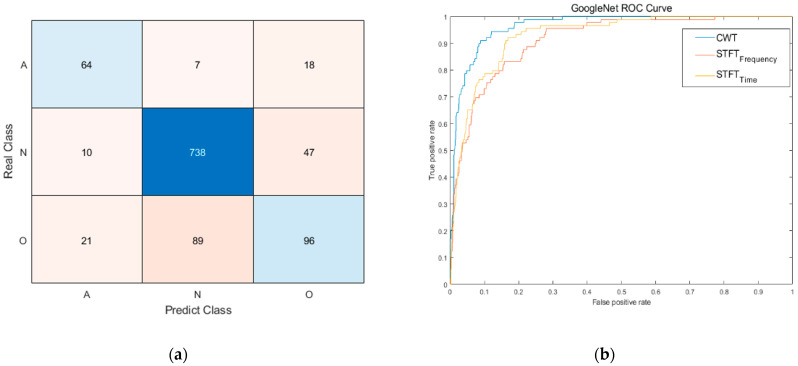
Test results from the selected model (GoogleNet with ADAM, CWT image set, 10^−4^ initial learn rate, minibatch size of 64, 10^−4^ of L2 regularization rate) (**a**) confusion matrix (**b**) ROC curve comparison with STFT image sets.

Table 6 shows the performance results for the test data. The four networks with three types of image sets were evaluated according to accuracy, F1 score, and AUC. GoogleNet showed very good accuracy, F1 score, and AUC compared to the other models throughout the whole image set. This clearly shows that GoogleNet demonstrates better performance in classifying arrhythmias than other networks. Since the accuracy of each network was similar, comparing the AUC was advantageous in finding a network with good performance, especially for the training dataset with different distributions for each class. Figure 10 shows the confusion matrix of GoogleNet with the CWT image set and the ROC curves of GoogleNet with the three image sets. GoogleNet using CWT shows a larger area than other networks, which enables a more stable prediction. These results show that the proposed CWT model had higher robustness in classifying ECG data into the AF, normal, and other rhythms classes compared to the STFT models. After comparing all the results, the selected network and its parameters are as follows:

- GoogleNet trained using ADAM optimizer.
- CWT-based ECG image set.
- Initial learning rate of 10^−4^.
- Minibatch size of 64.
- L2 regularization rate of 10^−4^.

## 6. Discussion

### 6.1. Preprocessing Results

The dataset used in the study was preprocessed before training. To understand the effects of preprocessing on training, the network was trained based on the data that had not been preprocessed, and its results were compared with the results based on the preprocessed data. To compare the results, the same data as the previously used test dataset was used, and their image set was created using CWT without preprocessing. To understand the contribution of each preprocess, three types of image sets were prepared: original data, data to which only normalization was applied, and data to which both normalization and noise removal were applied.

As a result of training with the original data without any preprocessing, an accuracy of 80.46% was obtained, and the data with only normalization came out to be 84.04%. The data that went through the two processes of normalization and noise removal showed an accuracy of 81.23%. Considering that the data that went through the whole preprocessing had an accuracy of 82.39%, it is noticeable that the accuracy of the data that only went through normalization is slightly higher. However, when comparing the classification results for the test data, the data that went through the whole preprocessing shows higher accuracy than the data that only went through normalization.

### 6.2. Comparison of the Original and Stitched Data

When looking at the confusion matrix of the test data, which was obtained through GoogleNet and shown in Table 7, the classification performance of AF is lower than that of normal. However, if the same test was performed with the original data without stitching, the classification performance for normal and AF is better represented as shown in Table 7 and Table 8. This is because the feature point (reference) of AF may be lost in the process of extracting cycles from data to acquire 2.5 s segments. Therefore, the original test data had an accuracy of 88.99%, but the scheme proposed in this study had a lower accuracy of 82.39%. However, this level of difference is expected to show better classification compared to the error that would occur when data were continuously received for 10 s during driving.

In the case of the other rhythms group, various inconsistent diseases, such as tachycardia, bradycardia, broad QRS complex, atrial flutter, and ventricular tachycardia, were included [28]. Therefore, in both cases, the accuracy for other rhythms is low, at 46% and 55%, respectively. In addition, other rhythms were often incorrectly predicted as normal. Because other rhythms include tachycardia and bradycardia, the heart rate of other rhythms is similar to the heart rate of normal. When generating the PhysioNet dataset, the data were visually classified. At this time, it is thought that other rhythms show lower classification compared to other labels because some of the data were changed to increase the number of data points [27]. Because of these increased data points, the classification results are not good even in the original dataset.

One of the key challenges in classifying driver arrhythmias during driving is accurately detecting them due to the noises generated during driving. The proposed ECG stitching scheme addresses this challenge by leveraging stable signal segments and stitching them together to generate a complete 10 s ECG signal. However, one limitation of this study is that the proposed ECG stitching scheme was only evaluated on a single dataset, which may limit its generalizability to real-time ECG signals. Future studies could address this limitation by evaluating the proposed scheme with real-time ECG signals and exploring its performance in different driver health monitoring systems.

## 7. Conclusions

In this paper, an ECG stitching scheme was proposed to detect arrhythmias of the driver during driving. The proposed scheme continuously extracts stable ECG signals while the driver drives a vehicle and classifies arrhythmias using CNN. To construct an ECG signal that is robust to noise generated during driving, this study proposed an algorithm that extracts a stable 2.5 s ECG segment and concatenates each segment into one 10 s full ECG signal.

Before the ECG stitching algorithm is applied, the data undergoes preprocessing consisting of normalization, noise section removal, and data flipping. For the ECG stitching, ECG data are divided into a series of cycles, and they are stored in a buffer. In order to extract the cycles from the ECG data, the R peaks, which are the central positions of each cycle, should be found first and then improved based on the TP intervals.

To apply the TP interval segmentation to a person with an arrhythmia, this study also considered the case where the P peak is not detected. In that case, the ECG data cannot be divided using the TP interval, so the P peak estimation is applied to the data.

In order to use CNN models for arrhythmia classification with stitched ECG data, each time series ECG signal was transformed via CWT, high-frequency resolution STFT, and high-time-resolution STFT. Finally, transfer learning is performed for classification using four CNN models: GoogleNet, SqueezeNet, ResNet, and DenseNet.

According to the classification results, compared to the other networks, GoogleNet showed the best performance according to classification accuracy, and the AUC or F1 scores were the best with the CWT image set. From the results, the classification accuracy was 82.39% for the stitched ECG data, which is 6% less than that of the original (unstitched) ECG data.

## Figures and Tables

**Table 1:** Stitching results by signal length.

Time	AF	Normal	Other Rhythms
2.0 s	43	773	228
2.1 s	47	780	221
2.2 s	59	798	203
2.3 s	66	798	203
2.4 s	85	805	196
2.5 s	89	795	206
2.6 s	88	794	207
2.7 s	79	792	209
2.8 s	81	766	235
2.9 s	63	773	228
3.0 s	70	747	254

**Table 2:** Confusion matrix of AF.

	AF	Normal	Other Rhythms
AF	TP	FN	FN
Normal	FP	TN	FP
Other Rhythms	FP	FP	TN

**Table 3:** Results: Optimizers.

	Network	Optimizer	Validation Accuracy	Test Accuracy	Validation Loss
CWT	GoogleNet	SGDM	77.46	79.36	0.5452
		**ADAM**	**81.95**	**82.39**	**0.4757**
	SqueezeNet	SGDM	76.8	78.44	0.564
		ADAM	80.1	80.55	0.4957
	ResNet	SGDM	77.8	79.08	0.5454
		ADAM	81.68	81.65	0.4864
	DenseNet	SGDM	78.07	78.35	0.551
		ADAM	79.51	79.82	0.516
STFT_F	GoogleNet	SGDM	72.33	75.96	0.6836
		**ADAM**	**79.22**	**80.28**	**0.554**
	SqueezeNet	SGDM	72.67	75.87	0.6553
		ADAM	78.9	78.81	0.5392
	ResNet	SGDM	73.89	76.88	0.7554
		ADAM	77.31	80.37	0.5711
	DenseNet	SGDM	75.78	78.26	0.6518
		ADAM	76.41	78.35	0.5881
STFT_T	GoogleNet	SGDM	73.94	77.89	0.6163
		**ADAM**	**79.29**	**80.64**	**0.5331**
	SqueezeNet	SGDM	75.58	78.53	0.6078
		ADAM	78.05	80.83	0.5382
	ResNet	SGDM	75.07	77.61	0.7499
		ADAM	76.95	80.18	0.5805
	DenseNet	SGDM	73.38	77.61	0.7184
		ADAM	75.63	79.54	0.5902

**Table 4:** Results: Networks.

	Network	Optimizer	Minibatch Size	V Acc	T Acc	V Loss
CWT	GoogleNet	ADAM	**64**	**81.95**	**82.39**	**0.4757**
			128	81.47	81.83	0.486
	SqueezeNet	ADAM	64	80.1	80.55	0.4957
			128	79.85	79.08	0.5118
	ResNet	ADAM	64	81.68	81.65	0.4864
			128	80.1	77.98	0.5197
	DenseNet	ADAM	64	79.51	79.82	0.516
			128	80.05	78.9	0.5437
STFT_F	GoogleNet	ADAM	**64**	**79.22**	**80.28**	**0.554**
			128	78.73	80.09	0.5573
	SqueezeNet	ADAM	64	78.9	78.81	0.5392
			128	78.83	79.08	0.5324
	ResNet	ADAM	64	79.18	80.37	0.5711
			128	79.27	79.36	0.6009
	DenseNet	ADAM	64	77.67	78.35	0.5881
			128	77.62	79.17	0.5793
STFT_T	GoogleNet	ADAM	**64**	**79.29**	**80.64**	**0.5331**
			128	79.19	80.18	0.5431
	SqueezeNet	ADAM	64	78.05	80.83	0.5382
			128	78.29	80.28	0.5371
	ResNet	ADAM	64	79.24	80.18	0.5805
			128	79.81	80.83	0.648
	DenseNet	ADAM	64	78.27	79.54	0.5902
			128	78.83	79.82	0.595

**Table 5:** Results: L2 regularization rates.

	Network	Optimizer	Minibatch Size	L2 Rate	V Acc	T Acc	V Loss
CWT	GoogleNet	ADAM	64	10^−4^	**81.95**	**82.39**	**0.4757**
				10^−4^	78.75	79.72	0.5101
				0	81.00	78.44	0.4783
	SqueezeNet	ADAM	64	10^−4^	80.10	80.55	0.4957
				10^−2^	79.95	82.52	0.5018
				0	**80.00**	**80.92**	**0.4903**
	ResNet	ADAM	64	10^−4^	81.68	81.65	0.4864
				10^−2^	79.07	80.46	0.5621
				0	**82.61**	**81.01**	**0.4790**
	DenseNet	ADAM	64	10^−4^	79.51	79.82	0.5160
				10^−2^	**81.47**	**82.02**	**0.4842**
				0	80.07	77.16	0.5348
STFT_F	GoogleNet	ADAM	64	10^−4^	**79.22**	**80.28**	**0.5540**
				10^−2^	77.56	79.45	0.5549
				0	79.10	80.00	0.5522
	SqueezeNet	ADAM	128	10^−4^	**78.83**	**79.08**	**0.5324**
				10^−2^	77.14	79.19	0.5692
				0	78.32	79.08	0.5609
	ResNet	ADAM	64	10^−4^	79.18	80.37	0.5711
				10^−2^	77.12	80.00	0.6089
				0	**79.34**	**81.47**	**0.6017**
	DenseNet	ADAM	128	10^−4^	76.90	79.17	0.5793
				10^−2^	**79.17**	**80.00**	**0.5499**
				0	78.17	79.54	0.5823
STFT_T	GoogleNet	ADAM	64	10^−4^	**79.29**	**80.64**	**0.5331**
				10^−2^	76.09	79.54	0.5814
				0	79.02	81.19	0.5433
	SqueezeNet	ADAM	64	10^−4^	**78.05**	**80.83**	**0.5382**
				10^−2^	77.00	80.09	0.5704
				0	78.32	79.17	0.5386
	ResNet	ADAM	128	10^−4^	**79.81**	**80.83**	**0.6480**
				10^−2^	77.44	78.81	0.6086
				0	78.71	80.73	0.7514
	DenseNet	ADAM	128	10^−4^	**78.83**	**79.82**	**0.5950**
				10^−2^	77.81	79.91	0.6029
				0	77.39	77.34	0.6134

**Table 6:** Acc, F1 score, AUC results based on stitched test data.

	Network	Acc	F1 Score	AUC
CWT	GoogleNet	82.39	0.5950	0.9650
	SqueezeNet	80.92	0.5066	0.9559
	ResNet	81.01	0.5646	0.9501
	DenseNet	82.02	0.5673	0.9579
STFT_F	GoogleNet	80.28	0.5141	0.9139
	SqueezeNet	79.17	0.5171	0.9269
	ResNet	81.47	0.5429	0.8992
	DenseNet	80.00	0.5018	0.9188
STFT_T	GoogleNet	80.64	0.5288	0.9292
	SqueezeNet	79.91	0.4996	0.9036
	ResNet	80.83	0.5342	0.9322
	DenseNet	79.82	0.5153	0.9240

**Table 7:** Confusion matrix of CWT GoogleNet.

Network		Stitched			Original (Non-Stitched)		
GoogleNet		Predicted Class			Predicted Class		
		A	N	O	A	N	O
True class	A	64	7	18	78	6	5
	N	10	738	47	2	778	15
	O	21	89	96	13	79	114

**Table 8:** Test results of CWT GoogleNet.

	Image Set	Acc	F1	AUC
GoogleNet	Stitched	82.39	0.5950	0.9650
	Original	88.99	0.7163	0.9869

## Data Availability

No new data were created or analyzed in this study. Data sharing is not applicable to this article.
